# Decoding community proximity discourse: A mixed-methods comparative analysis of online local and national newspapers in Romandy, Switzerland

**DOI:** 10.1371/journal.pone.0328059

**Published:** 2025-08-01

**Authors:** Victor Bros, Daniel Gatica-Perez

**Affiliations:** 1 Idiap Research Institute, Martigny, Switzerland; 2 EPFL, Lausanne, Switzerland; Universidade de Santiago de Compostela, SPAIN

## Abstract

This paper presents a mixed-method approach to analyzing news media, combining quantitative linguistic metrics with qualitative discourse frameworks. We first extract linguistic features such as quotations, readability levels, and lexical richness, then perform named entity recognition and topic modeling. To add depth, we apply Fairclough’s model of critical discourse analysis—highlighting social and cultural contexts—together with Goffman’s frame analysis of social behavior, enabling a systematic comparison of narrative strategies and community engagement. We provide new evidence on how local and national papers diverge not only in content, but also in temporal framing, participatory practices, and the construction of proximity. We validate this pipeline by comparing local and national newspapers in Romandy, Switzerland, a media landscape where local press agencies currently face intense economic pressures and widespread layoffs. Our findings reveal notable divergences in narrative construction and audience engagement: local outlets focus on fostering a sense of community and direct connection with readers, whereas national outlets employ more wide-ranging, sophisticated storytelling to appeal to a broader audience. By synthesizing quantitative and qualitative insights, our study moves beyond descriptive comparison to show how distinct editorial logics shape identity and democratic participation in a transforming media landscape. The study’s integrated analytical framework underscores the importance of a comprehensive, multi-level perspective on media strategies and audience dynamics, particularly in an era of rapid editorial and economic transformations.

## 1 Introduction

### Background and context

The local press has historically played a significant role in reflecting regional interests. Established in the mid-19th century during a transformative period in the media landscape, particularly in Victorian England, the abolition of newspaper taxes facilitated the proliferation of affordable information and decentralized news consumption [1–3]. This shift not only challenged the dominance of the metropolitan press, but also symbolized an emancipation from centralization.

Today, the local press continues to connect democratic institutions and communities [4]. Despite structural changes and economic challenges [5,6], it remains a key component of information dissemination. However, the closure of local titles and the decline in readership have led to the emergence of so-called “information deserts”, where communities lack access to diverse and locally relevant news. This phenomenon, often referred to as “news deserts”, has been documented across the United States, Europe, and other regions, and is characterized by the absence or severe reduction of original local journalism [7–9].

Research shows that news deserts are not only a rural or peripheral issue, they can also affect urban and suburban areas, and their boundaries are shaped by economic, technological, and policy factors [10,11]. In the UK, for example, recent mapping efforts have identified both absolute and relative news deserts, with some local authority districts having no dedicated local news outlets at all [12]. The consequences of these deserts are far-reaching: communities without robust local news experience lower civic engagement, reduced government accountability, and increased vulnerability to misinformation and polarization [13–15]. The decline of local journalism also weakens its traditional role as a political institution and a source of community identity [16,17].

The causes of news deserts are complex, including the collapse of traditional business models, the dominance of digital platforms, and the concentration of media ownership, all of which have contributed to the erosion of proximity between journalists and their communities [12,18]. While digital transformation offers new opportunities for audience engagement and participatory journalism, it also presents challenges in sustaining the financial and civic value of local news [19–22]. In many cases, social media and online platforms have not fully replaced the institutional functions of local news, sometimes even amplifying misinformation and polarization [14,23].

At the same time, the local press remains valuable for maintaining social cohesion and democratic engagement. Skepticism towards national media has sparked renewed interest in local journalism, which is often perceived as a “good neighbor” focusing on community life and fostering connections within communities [24–27]. The ability of local news to connect democratic institutions and communities is especially important in contexts where media fragmentation and polarization shape user perceptions [28]. In Switzerland, for example, users engage with a spectrum of media, from traditional outlets to alternative sources, shaped by the political information environment.

To adapt to the changing landscape, local press actors have transitioned to digital formats and experimented with new business models, such as paywalls, memberships, and community partnerships [19,20,29,30]. However, the tension between commercial imperatives and the democratic role of local journalism remains a central concern [16,17]. Traditional revenue from print advertising is dwindling, and digital revenues are lower. Consequently, transforming the business model toward attracting more online users becomes necessary [31,32]. This transition involves competition with digital giants and adaptation to new consumption habits, particularly among younger demographics who prefer digital platforms [33–35]. While traditional media are increasingly overlooked by youth, their interest in news persists: they seek accessible information.

Moreover, the media landscape in Switzerland has also undergone significant transformation. The rise of free newspapers targeting supra-regional audiences and a shift toward advertising-centric business models have reconfigured the press ecosystem [36]. As a result, local news coverage has diminished in favor of broadly appealing content [37,38]. The resulting contraction of local journalism raises concerns about democratic engagement and the weakening of local journalism’s role. This trend is exemplified by recent large-scale job cuts and the closure of printing plants at Tamedia, one of Switzerland’s major publishers, which have been widely reported in the press [39–41]. Recognizing the need to preserve local journalism, several initiatives aim to revitalize local news through accessible information and community-focused reporting [22,30].

Scholarly work also indicates that structural factors, including ownership concentration and economic models, substantially steer the diversity of news coverage. For instance, concentrated ownership can limit coverage variation and potentially marginalize community-specific content [42]. Such insights underscore the challenges in balancing commercial viability with the community-oriented mandate of local newspapers.

Within this evolving environment, distinctions between local and national news have become particularly salient. In the Swiss context, clarifying these differences is essential for understanding how each outlet approaches its audience. This paper examines these differences through a comprehensive analytical framework that combines computational approaches and qualitative methodologies. By focusing on local newspapers and their national counterparts in Romandy, the study reveals how local-oriented content coexists with more generalized national coverage.

### Research questions and contributions

In this paper, we address two research questions:

- RQ1 : How can a mixed-methods analytical pipeline, combining computational linguistic metrics and qualitative discourse analysis, be systematically designed and applied to uncover the discursive construction of community proximity in digital news content?
- RQ2 : In what ways do local and national newspapers in Romandy, Switzerland, differ in their narrative strategies, thematic focus, and engagement with community identity, as revealed by this integrated approach? How do these differences reflect the distinct roles and societal functions of local versus national media, as interpreted through critical discourse and frame analysis?

By integrating readability and lexical metrics with qualitative tools for discourse analysis, we distinguish signals of local identity from national-level narratives that rely on more generalized frames. Hence, in RQ1, we ask how such a combined methodological approach reveals local-oriented strategies in comparison to national coverage. In RQ2, we focus specifically on how those strategies manifest in different content categories, measuring the degree of community emphasis, direct citation of local figures, and framing scope. These questions are motivated by the need to move beyond descriptive comparisons, aiming instead to explain how and why local and national media construct proximity and community in distinct ways. By explicitly linking computational indicators to qualitative interpretation, our approach provides a more comprehensive understanding of media strategies and their implications for democratic engagement.

We make four main contributions relevant to open, interdisciplinary media research. (1) We curated a dataset of local newspaper articles from Romandy, serving as an empirical basis for future investigations of local news coverage. (2) We designed and documented a reproducible pipeline that combines advanced computational text analytics with grounded qualitative discourse analysis. (3) We conduct a comparative study that underscores the distinct editorial approaches used by local and national outlets, advancing our understanding of how coverage scale shapes media content and audience engagement. (4) We publicly release our code and dataset, thereby promoting methodological transparency, reproducibility, and opportunities for further research on local news dynamics.

The remainder of this paper is structured as follows: Sect 2 reviews the existing literature on local vs. national news and analytical methods in journalism. Sect 3 details the methodology, including data collection, preprocessing, quantitative analysis, topic modeling, and qualitative analysis. Sect 4 presents the results of the study, followed by a discussion in Sect 5. Finally, Sect 6 concludes the paper and suggests areas for future research.

## 2 Literature review

The dynamics between local and national news outlets significantly influence public perception, democratic engagement, and the broader media landscape. This literature review examines comparative studies of local and national news, the application of computational methods in journalism, theoretical frameworks for news analysis, and the implications for democratic participation and public opinion. By examining these areas, we situate our study within the existing literature and identify gaps that our research seeks to address.

### 2.1 Editorial proximity and community orientation in local and national news

Comparative research on local and national news has revealed both convergences and divergences in editorial strategies, content, and democratic impact. In French-speaking Europe, Pignard-Cheynel and Amigo [43] document how local news organizations have implemented participatory practices to strengthen audience connections, reflecting a broader trend toward engagement and responsiveness. In Switzerland, Kübler and Goodman [44] show that when newspaper markets align with municipal boundaries, voter turnout increases, suggesting that robust local media environments can support democratic participation.

The challenges facing local journalism are not unique to Switzerland. Mota [15] highlights how the collapse of traditional business models and the rise of digital platforms have eroded the proximity between journalists and their communities, threatening the civic and democratic functions of local news. In the UK, Matthews [16] argues that the commercial imperatives of the provincial press often conflict with its role as a community watchdog, and calls for new models that prioritize editorial quality and community benefit. Pickard [17] further critiques the structural crisis of commercial journalism in the US, advocating for systemic reforms to reimagine journalism as essential democratic infrastructure.

The resilience and adaptation of local newspapers have also been studied in the US context. Radcliffe and Ali [19] find that small-market newspapers, while facing slow digital adoption, have shown notable resilience by focusing on ultra-local content and experimenting with diversified revenue streams. In Norway, Reiners *et al*. [20] analyze how local newspapers use paywalls to balance the needs of print subscribers and digital audiences, while Vara-Miguel *et al*. [21] show that local and regional outlets in Spain often rely on public and private grants, highlighting the financial vulnerability of smaller-scale media.

Content analyses comparing local and national news coverage have produced nuanced findings. Lawlor [45] observes that local and national newspapers in Canada and the UK often share similar editorial narratives when framing immigration, while Cooper *et al*. [23] demonstrate that regional Australian newspapers can challenge dominant national frames by presenting more positive and humane stories about refugees. In the US, studies of high-profile events such as the Aurora shootings [46] and the Jena Six controversy [47] reveal that local outlets tend to emphasize community impacts and human-interest frames, whereas national outlets are more likely to focus on societal or moral dimensions. In Hong Kong, research by Lee and Chan [48] shows that geographic distance influences framing strategies, with local news relying more on community voices and national news emphasizing institutional perspectives.

The interplay between national and local agendas is further explored by Funk and McCombs [49], who find that while national media can influence local coverage through agenda-setting, local community characteristics play a crucial role in shaping how issues are framed. In Sweden, Nord [50] finds that local journalism’s watchdog function is limited, with few articles containing substantive criticism and journalists often outsourcing critical perspectives to external sources. Recent mapping of the UK news landscape by the Public Interest News Foundation [8] underscores the impact of ownership concentration and the persistence of news deserts, which further constrain the diversity and robustness of local news.

Overall, these studies suggest that the relationship between local and national news is shaped by a complex interplay of editorial strategies, business models, and community context. Local media can both reinforce and diverge from national agendas, and their capacity to serve democratic functions depends on structural, economic, and cultural factors.

### 2.2 Computational approaches to news content analysis

The rise of computational methods has transformed the study of news content, enabling researchers to analyze large-scale corpora and uncover patterns that would be difficult to detect manually. Topic modeling, for example, has been widely used to identify predominant themes in news coverage. Griciūtė *et al*. [51] track the evolution of COVID-19 topics in Swedish newspapers, revealing how public health, scientific, and economic issues shifted over time. Yang *et al*. [52] extend these techniques to short texts, such as headlines and tweets, by incorporating crowdsourcing to improve topic coherence.

Machine learning approaches have also advanced narrative analysis. Jones *et al*. [53] demonstrate that transformer-based models like BERT can approximate human ratings in evaluating the macrostructure of news narratives, suggesting that computational tools can complement qualitative analysis. Lee *et al*. [54] use topic modeling to show that Korean news coverage of nursing legislation is dominated by political and institutional conflict frames, rather than substantive policy discussion.

Framing analysis has benefited from computational innovation as well. Guo *et al*. [55] introduce Open Framing AI, a framework that leverages multilingual deep learning to detect frames with minimal manual annotation, making systematic framing analysis more accessible. Barrio [56] and Eisele *et al*. [57] further illustrate how large language models and supervised approaches can capture nuanced frames in news stories, often outperforming semi-supervised methods.

These computational advances have expanded the analytical toolkit for journalism studies, but their findings are most meaningful when interpreted through robust theoretical frameworks that account for the social and cultural dimensions of news production.

### 2.3 Theoretical frameworks: Discourse and frame analysis

Critical Discourse Analysis (CDA) and frame analysis provide essential lenses for interpreting the deeper meanings embedded in news texts. Fairclough’s CDA situates language within broader social and ideological contexts, enabling researchers to uncover how word choices and discursive strategies reflect editorial stances and power relations. Hermawan and Hamdani [58] apply Fairclough’s model to online news about refugees, revealing how discourse can reinforce or challenge stereotypes. Bednarek and Caple [59] further develop CDA by showing how news values such as proximity and eliteness are constructed through linguistic and multimodal resources, shaping what is considered newsworthy.

Frame analysis, rooted in Goffman’s work, examines how news outlets structure reality by highlighting certain aspects of events. Linström and Marais [60] provide a methodological guide for qualitative frame analysis, emphasizing the interpretive process of identifying frames through word choice, metaphors, and sources. Spradlin and Givens [61] compare local and national newspapers’ coverage of climate change, finding that local outlets often use less partisan frames and focus more on human-interest stories. Ferrer-Conill *et al*. [62] show that even visual elements can influence how news is framed, blurring the boundaries between editorial and sponsored content.

Recent studies have also highlighted the importance of integrating corpus-assisted and multimodal analysis in news discourse research [63,64]. These approaches allow for a more systematic examination of how news values and frames are constructed and contested across different media contexts.

### 2.4 Implications for democratic engagement and public perception

The decline of local journalism has significant implications for democratic engagement and public perception. Ellger *et al*. [65] find that the closure of local newspapers in Germany leads to increased electoral polarization, as citizens turn to more partisan national tabloids. In the US, Moskowitz [66] shows that the erosion of local reporting contributes to the nationalization of elections, with voters more likely to align their choices with national partisan trends in the absence of local news. More broadly, Earle and Hodson [67] demonstrate that the tone and framing of news media, regardless of scale, can significantly shape sociopolitical attitudes on issues such as immigration and gun control, reinforcing the importance of how both local and national outlets construct their narratives.

Civic engagement is also affected by the availability of local news. Shaker [13] demonstrates that newspaper closures in US cities are associated with declines in civic participation, while Ha *et al*. [68] highlight the role of local and national news in shaping political efficacy, especially among young and undecided voters. The experiences of young people across Europe, as explored by Cushion *et al*. [69], reveal that local news can foster critical citizenship and community identity, even in contexts of social exclusion.

The literature underscores the importance of robust, original, and genuinely local news in supporting democratic participation and meeting critical information needs [9]. As local journalism contracts, understanding the distinctions between local and national news becomes increasingly important for assessing the health of democratic societies.

### 2.5 Addressing the research gap

Despite the literature on local vs. national news variations and the proliferation of computational approaches, there remains a shortage of research that methodically integrates quantitative and qualitative analyses in assessing local newspapers relative to their national counterparts. Furthermore, Romandy’s French-language media has received limited attention. This study contributes to closing those gaps by applying a mixed-method pipeline to compare local and national newspapers in Romandy, Switzerland. By combining readability and lexical metrics with a grounded qualitative approach rooted in Fairclough’s discourse model and Goffman’s framing theory, the research offers a structured understanding of local-oriented journalistic practices in contrast to more generalized national news narratives.

## 3 Methodology

### 3.1 Definition of local and national news

Inspired by the definitions used in Norwegian news studies [70], we developed a Swiss-specific definition, considering the fragmentation into cantons and multiple language regions. We classified *Le Nouvelliste*, *Arc Info*, and *La Côte* as “Local News” because each title primarily serves a single canton or region. We labeled *La Tribune de Genève* (TDG), 24*heures* (24h), and 20*minutes* (20min) as “National News”, in view of their wider distribution across the Romandy area. This classification differs somewhat from the outlets’ self-definitions (e.g. TDG or 24h see themselves as regionally focal), but it allowed us to compare newspapers targeting a narrower local audience with those reaching a wider readership.

This analytical distinction is supported by recent developments in the editorial strategies of TDG and 24h. Both outlets have undergone a shift under the Tamedia group, extending their editorial focus and newsroom allocation beyond single cantons to address the broader Romandy public. For instance, recent newsroom reports have highlighted an internal concern at 24h regarding the diminishing coverage of Vaud-specific news, as staff are increasingly reassigned to supra-cantonal topics [71]. Furthermore, these newspapers now prominently feature national and international sections on their homepages, in contrast to the more locally anchored content and structure observed in the other local titles in our dataset. Accordingly, our definition operationalizes “local” news outlets as those whose editorial mission, organization, and coverage remain primarily focused on a single canton or region, while classifying as “national” those titles adopting a supra-cantonal strategy, even if they retain regional branding.

### 3.2 Data collection

We collected news articles published between June 2019 and June 2022 from two main sources: an internal archive provided by ESH Médias for local newspapers and the CCNews dataset [72] for national newspapers. We stored the articles with metadata (title, headline, text content, authors, date) and, where available, editorial tags. Basic cleaning steps included removing text artifacts (e.g. HTML tags) and formatting errors.

Furthermore, the selected timeframe for this study, June 2019 to June 2022, was determined by the availability of high-quality, consistently archived news articles from both local and national outlets. This period ensures a robust and balanced comparison, as it represents the largest window for which comprehensive and reliable data could be extracted across all sources. Earlier or later periods were excluded due to inconsistencies or gaps in the archives, which would have compromised the comparability and integrity of the dataset.

#### 3.2.1 Local news dataset.

The local news dataset was obtained from the ESH Médias platform, which operates three French-language daily local newspapers in Romandy, Switzerland: *Le Nouvelliste* (Valais), *ArcInfo* (Neuchâtel), and *La Côte* (Vaud). These newspapers focus on reporting news from their respective cantons, covering national news only when significantly relevant.

Despite the decline in print media, these newspapers have maintained stable readerships until recent years. Circulation is approximately 45,000 printed copies daily for *Le Nouvelliste* (100,000 readers), 32,000 copies for *ArcInfo* (55,000 readers), and under 10,000 copies for *La Côte* (unavailable readership data).

Insights from our contact with the editorial teams provided valuable context. *Le Nouvelliste* employs 50 permanent journalists, *ArcInfo* 35, and *La Côte* 15, suggesting staff size correlates with activity levels. Few journalists contribute to more than one newspaper. Post-COVID-19, the newspapers have evolved their approach to strengthen local focus and present local news uniquely, enhancing visibility within local communities. It is estimated that 95% of online articles also appear in print. According to our contact, automation is minimal, used only for article recommendations on the website.

We collected articles published between June 2019 and June 2022, focusing on this period due to earlier data inconsistencies. For each article, we gathered the title, headline, content, authors, date, and journalist-annotated tags. Table 1 summarizes the dataset.

**Table 1:** Article counts for each local newspaper. Numbers in parentheses indicate unique articles, with duplicates across newspapers counted once.

Local Newspapers (June 2019 - June 2022)			
Le Nouvelliste	La Côte	Arc Info	Total
43,393	38,283	48,479	130,155
(21,581)	(12,618)	(22,830)	(83,243)

#### 3.2.2 National news dataset.

From the CCNews dataset scraped by CCbot, we collected national news from 20min, 24h, and TDG, focusing on June 2019 to June 2022. These sources were selected based on their reliability, given the variable quality in the CCNews collection. For each article, we collected the title, headline, content, authors, and date. A summary of the dataset is shown in Table 2.

**Table 2:** Article counts for each national newspaper. Numbers in parentheses indicate unique articles, with duplicates across newspapers counted once.

National Newspapers (June 2019 - June 2022)			
20min	24h	TDG	Total
72,252	66,533	60,629	199,414
(71,212)	(18,842)	(13,050)	(150,545)

#### 3.2.3 Data access and availability.

The local news articles were obtained through a partnership with ESH Médias and are stored at the Idiap Research Institute. While the dataset is not directly downloadable, it is available to academic researchers upon request with a brief application to the data management team at Idiap. This procedure is in place to ensure that the data is used exclusively for research purposes, in accordance with the agreement with the press agency. Requests are typically processed and access granted within 1-2 business days, and we have found this process to be efficient and not a barrier to scholarly use. The national news dataset is publicly available and can be accessed without restriction.

### 3.3 Contextual overview of the sample period

The period covered by our dataset (June 2019–June 2022) was marked by a rich diversity of events and recurring themes that shaped news coverage in Romandy and Switzerland. While the COVID-19 pandemic formed a significant backdrop, affecting public life, event organization, and media priorities, news dynamics in this period were also driven by a variety of political, cultural, and social developments.

Major political milestones included the Swiss federal elections, ongoing debates over Switzerland’s bilateral relationship with the European Union, and high-profile referendums such as the ${CO}_{2}$ law. International events such as the US presidential election, the *Tokyo Olympics*, and the *FIFA World Cup* also received substantial attention, often with a local angle reflecting the participation or interests of Romandy communities.

Cultural life in Romandy remained vibrant, with flagship events such as the *Montreux Jazz Festival*, the *Fête de l’Escalade* in Geneva, and the *Fête des Vignerons* in Vevey, an iconic winegrowers’ festival held only once a generation, serving as focal points for local identity and media coverage. The launch of the *Léman Express*, the cross-border commuter rail network, represented a major development in regional mobility and urban life. Annual traditions, such as the Christmas markets and the feminist strike each June, continued to mobilize communities and generate sustained media interest, even as their formats adapted to changing circumstances.

National and local sports, including football, hockey, and basketball leagues, provided a continuous thread of coverage, reflecting both community engagement and the broader rhythms of Swiss sporting life. Environmental and social issues, such as debates over animal rights (notably the management of wolves in alpine regions), climate activism, and gender equality, were also prominent in both local and national news agendas.

Overall, this period was characterized by a dynamic interplay between local events, national developments, and global trends, all of which are reflected in the thematic diversity and temporal fluctuations of our news sample. This high-quality sample ensures that our analysis captures the complexity and richness of the Romandy media ecosystem during a time of significant change.

### 3.4 Quantitative analysis

The quantitative analysis compares local and national news using metrics such as publication frequency, article length, number of quotes, named entity recognition, readability, and lexical richness. These metrics were implemented using Python libraries to ensure a reproducible analysis framework.

#### 3.4.1 Content metrics.

We assessed basic content characteristics of each news outlet from June 2019 to June 2022 by calculating:

- **Publication Frequency**: Number of articles published, indicating content volume and ensuring consistency in data comparison.
- **Article Length**: Number of words per article, reflecting the depth and detail of coverage.
- **Quotation Count**: Number of quotes per article, serving as a proxy for journalistic practices and the degree of closeness between news reporting and its audience.

#### 3.4.2 Named entity recognition.

For Named Entity Recognition (NER), the GLiNER model [73] was selected. This compact model uses a bidirectional transformer encoder for efficient parallel entity extraction and can prompt categories to specific news topics.

We prompted the model to recognize the following entity categories relevant to the analysis: (1) **Person**, (2) **Location**, (3) **Event**, (4) **Organization**, and (5) **Issues**. These categories were identified as relevant for comparing how local and national news portrays individuals, groups, and places.

#### 3.4.3 Readability metrics.

Article readability was assessed using standard English metrics adapted for French texts, focusing on relative comparisons:

- **Flesch Reading Ease**: Based on sentence length and syllable count.
- **Gunning-Fog Index**: Estimates years of education needed to understand the text, based on the proportion of complex words.
- **Coleman-Liau Index**: Uses characters per word and words per sentence.
- **Flesch-Kincaid Grade Level**: Translates the Flesch Reading Ease score into a school grade level with modified weights.
- **Automated Readability Index (ARI)**: Based on characters per word and words per sentence.

To adapt the Flesch Reading Ease metric to French, the Kandel-Moles formula [74] was used. An inverse transformation was applied to standardize the metrics, ensuring higher scores indicate increased text complexity and reading skill required.

#### 3.4.4 Lexical richness metrics.

Lexical richness was evaluated using the lexical_richness library [75]. The following standard metrics were computed:

- **Type-Token Ratio (TTR)**: The ratio of unique words (types) to the total number of words (tokens).
- **Herdan’s C**: A measure of lexical diversity that accounts for text length.
- **Dugast’s U**: Another measure of lexical diversity, similar to Herdan’s C.
- **Yule’s K**: A measure of lexical richness that considers the frequency distribution of words.
- **Measure of Textual Lexical Diversity (MTLD)**: A measure that is less sensitive to text length.
- **Hypergeometric Distribution Diversity (HDD)**: A measure that estimates the probability of encountering new words in a text.
- **Moving-Average Type-Token Ratio (MATTR)**: A sliding window approach to calculate TTR over segments of the text.

These metrics account for text length and word frequency distribution, providing a comprehensive analysis of lexical richness.

Overall, the quantitative analysis provides a comprehensive set of metrics to characterize and compare the local and national news in Romandy, offering insights into publication frequency, article length, number of quotes, readability, lexical richness, and the types of entities discussed.

### 3.5 Topic modeling

We employed the BERTopic framework [76] to extract and compare major topics between local and national news outlets. This process involved generating sentence embeddings, reducing dimensionality, clustering, and interpreting the resulting clusters.

#### 3.5.1 Embedding model.

We utilized the *Sentence-CamemBERT-Large* embedding model [77] to generate high-quality sentence embeddings for the French language. This model is based on the pre-trained *facebook/camembert-large*, and Siamese BERT-Networks with the *sentence-transformers* library [78], and further refined on the STS-B (Semantic Textual Similarity Benchmark) dataset. It provides state-of-the-art French sentence embeddings suitable for semantic search and analysis, representing the content and semantics of sentences as mathematical vectors for deeper textual understanding.

#### 3.5.2 Dimensionality reduction.

To prepare the embeddings for clustering, we applied the Uniform Manifold Approximation and Projection (UMAP) algorithm for dimensionality reduction, effectively preserving the data’s global structure while simplifying complexity. Subsequently, Hierarchical Density-Based Spatial Clustering (HDBSCAN) was employed for clustering. HDBSCAN extends DBSCAN to identify clusters of varying densities, facilitating efficient and interpretable clustering and allowing comparison of the hierarchical structure of topics between local and national news.

#### 3.5.3 Cluster interpretation.

Finally, we interpreted the resulting clusters using a method inspired by KeyBERT [79], extracting the most representative keywords for each cluster to summarize the topics. Keywords were selected based on their relevance and frequency within the cluster, aiding in interpreting the underlying themes. This approach allowed us to compare the organization and hierarchy of topics between local and national news, providing insights into differences in content and focus at different scales.

### 3.6 Qualitative analysis

The qualitative analysis aimed to provide deeper insights into the content and framing differences between local and national news. Since computational methods usually consider each article as a single data point, human qualitative analysis fills this gap by linking articles through contextual knowledge. Therefore, we conducted this analysis using a combination of Fairclough’s and Goffman’s content analysis frameworks, with content coding following Grounded Theory principles.

#### 3.6.1 Selection of categories and topics.

To structure the qualitative analysis, we first identified six broad themes commonly covered in both local and national news in Switzerland. These themes are:

1. Sports
2. Environment, Animals & Climate
3. Political Scenes
4. Business & Economy
5. Cultural Events
6. Justice & *Faits Divers*

Using the topics generated by the BERTopic model, we selected specific topics falling under each of these six themes. This selection ensured that the qualitative analysis focused on relevant and representative content within each theme.

#### 3.6.2 Content analysis frameworks.

The content analysis was guided by two established frameworks:

- **Fairclough’s Three-Dimensional Framework**: This model views language as social practice across three dimensions—textual analysis, discursive practice, and social practice [80]. It was used to examine how language constructs social realities, focusing on vocabulary, grammar, cohesion, and text structure.
- **Goffman’s Frame Analysis**: This framework explores how experiences are organized through interpretive frames [81]. It assisted in analyzing how news stories are presented and which perspectives are highlighted, focusing on selection and emphasis, metaphors, and narrative structure.

#### 3.6.3 Grounded theory for content coding.

The content coding process followed the principles of Grounded Theory, involving iterative coding and constant comparison to identify emerging themes and patterns [82]. The steps involved in the coding process were as follows:

1. **Open Coding**: Initial coding of the text to identify key concepts and categories.
2. **Axial Coding**: Linking categories and subcategories to form a coherent structure.
3. **Selective Coding**: Refining and integrating categories to develop core themes.

As the qualitative analysis was conducted by one annotator (the first author), measures were taken to ensure the reliability and validity of the coding process. The annotator maintained a detailed coding journal to document decisions and reflections, and periodic reviews were conducted to ensure consistency. A more detailed description of the methodology is available in the Appendix.

#### 3.6.4 Integration with quantitative and topic modeling results.

The qualitative findings were integrated with the quantitative metrics and topic modeling results to provide a comprehensive analysis. This integration allowed for a multi-faceted understanding of the differences between local and national news, highlighting both the quantitative and qualitative dimensions of the content. Overall, the qualitative analysis provided valuable insights into the nuances of news content and framing, complementing the quantitative and topic modeling analyses.

### 3.7 Code availability

To promote transparency and facilitate reproducibility of our research, the complete code for the analysis pipeline used in this study is available on GitLab. This repository includes scripts for the quantitative analysis, and the topic modeling, along with detailed instructions for replicating the results presented in this paper. The repository can be accessed at: https://gitlab.idiap.ch/socialcomputing/proximity-discourse-news-romandy.

## 4 Results

### 4.1 Quantitative findings

This section presents the quantitative differences observed between local and national news outlets in Romandy. The following subsections detail the findings for each metric, supported by statistical tests.

#### 4.1.1 Publication frequency.

**Fig 1:**
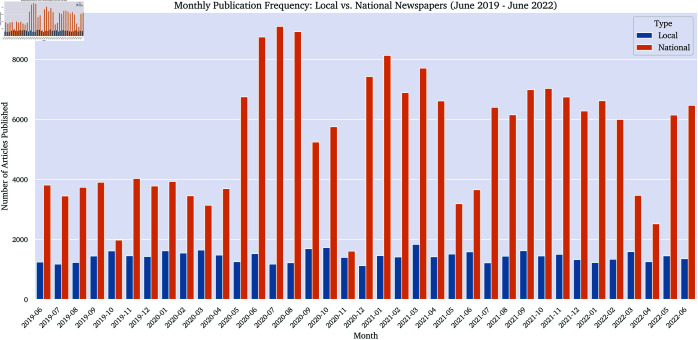
Publication frequency (monthly) of local vs. national news outlets from June 2019 to June 2022.

The frequency of publication differs between local and national news outlets. Local news outlets (Le Nouvelliste, Arc Info, La Côte) present a lower overall publication frequency compared to national news outlets (20min, TDG, 24h), as shown in Fig 1. While the local sources display consistent data collection from June 2019 to June 2022, certain gaps are visible for national sources, often due to website restrictions that temporarily blocked the CCBot from accessing content (e.g. November 2020).

The lower publication frequency of local news outlets compared to national titles reflects the resource constraints and narrower audience focus typical of local journalism. This pattern is consistent with the literature on the economic pressures facing local media [7], but it also highlights the challenge of maintaining a robust local news ecosystem in the face of national and digital competition. The steadiness of local publication, despite these constraints, may indicate a commitment to community service and continuity, a theme that will be further explored in the qualitative analysis.

#### 4.1.2 Article length.

**Fig 2:**
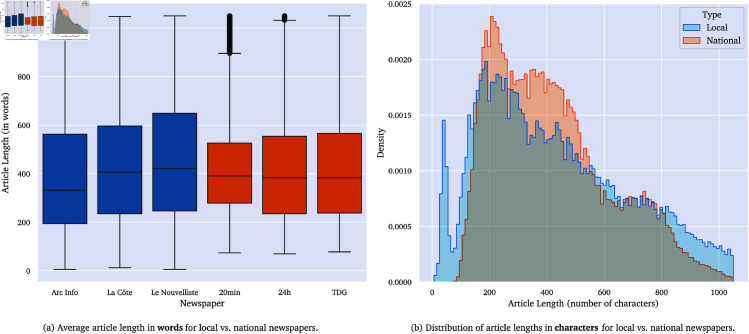
Comparison of article lengths (a) Box plots and (b) Histogram for local vs. national news outlets.

Fig 2 presents a comparison of article lengths based on both word and character counts. On average, local news articles are longer than their national counterparts. While the overall distributions are similar, several very short articles are found in both corpora, often stemming from timeline pages that contain only headlines.

The finding that local news articles are, on average, longer than national ones is notable. While national outlets may prioritize brevity and rapid news cycles to cater to a broader, more heterogeneous audience, local outlets appear to invest in more detailed storytelling. This supports the idea that local journalism seeks to provide context and depth on issues of direct relevance to its community, reinforcing its connective and explanatory role [19]. The longer format may also facilitate the inclusion of more voices and perspectives, as seen in the higher quote counts.

#### 4.1.3 Number of quotes.

**Fig 3:**
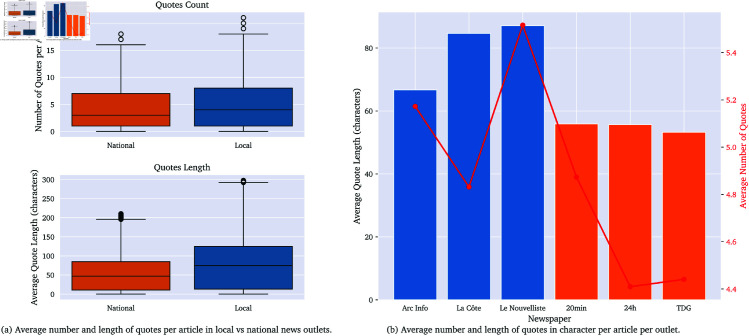
Comparison of average number of quotes (a) Box plots and (b) Histogram for local vs. national news outlets.

Local articles generally contain more direct quotes than national articles, as shown in Fig 3. This difference, found to be significant in the statistical tests, supports the notion that local outlets aim to foster a closer relationship with their readership, with a greater emphasis on direct speech from sources. Table 3 summarizes the metrics for both quote count and quote length.

**Table 3:** Comparison of quotes metrics between national and local articles.

Metric	National	Local
Quotes Count		
Mean	5.08	5.70
Standard Deviation	5.44	6.12
Minimum	0	0
Maximum	77	143
Quotes Length		
Mean	64.30	84.00
Standard Deviation	72.55	84.93
Minimum	0	0.0
Maximum	3,902	2,578.0
Statistical Tests		
T-statistic Quotes Count	-21.39	
P-value Quotes Count	*p*<0.001**	
T-statistic Quotes Length	-48.84	
P-value Quotes Length	*p*<0.001**	

The significantly higher number of direct quotes in local news articles suggests a deliberate editorial strategy to foreground community voices and foster a sense of proximity. This aligns with the notion of local news as a participatory platform [43], where readers may recognize themselves or their neighbors in the coverage. The use of direct speech not only personalizes the news but also strengthens the perceived legitimacy and trustworthiness of local outlets, potentially counteracting trends of declining civic engagement in news deserts.

#### 4.1.4 Named entity recognition.

**Fig 4:**
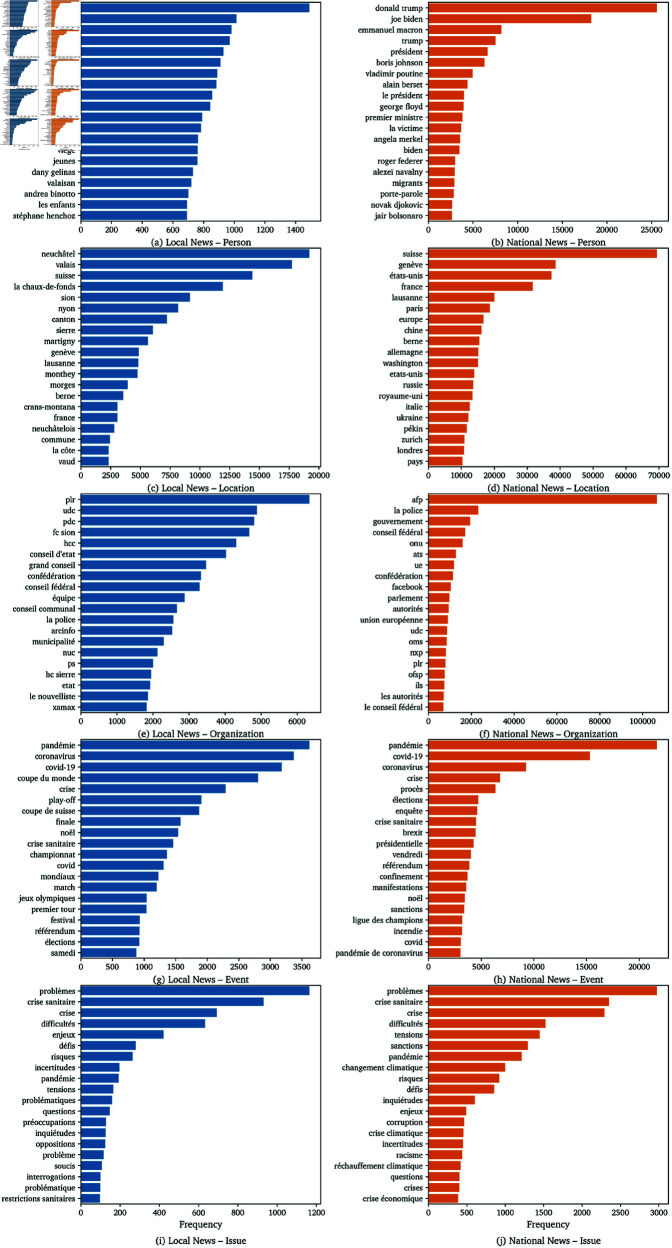
Comparison of Top NERs in local news vs national news across the 5 Categories.

Using the GLiNER model, we identified named entities (Person, Location, Event, Organization, Issues) in both local and national news. Fig 4 illustrates the distribution of top named entities for each category. Local news focuses on local personalities and places, for example featuring names like *Lucas Vuitel* or cities such as *Neuchâtel*, *La Chaux-de-Fonds*, and the canton of *Valais*. National news highlights more widely recognized figures and broader locations, such as *Donald Trump* or *Suisse*, *Genève*, *États-Unis*.

The prominence of local personalities, places, and organizations in local news, contrasted with the broader and more nationally or internationally recognized entities in national news, underscores the distinct audience orientation of each media tier. Local news’ focus on hyper-local entities reinforces its role in constructing and maintaining community identity, while national news’ broader scope reflects its function as a mediator of national discourse. This finding supports the theoretical expectation that proximity is not just geographic, but also discursive and symbolic [59].

#### 4.1.5 Readability metrics.

**Fig 5:**
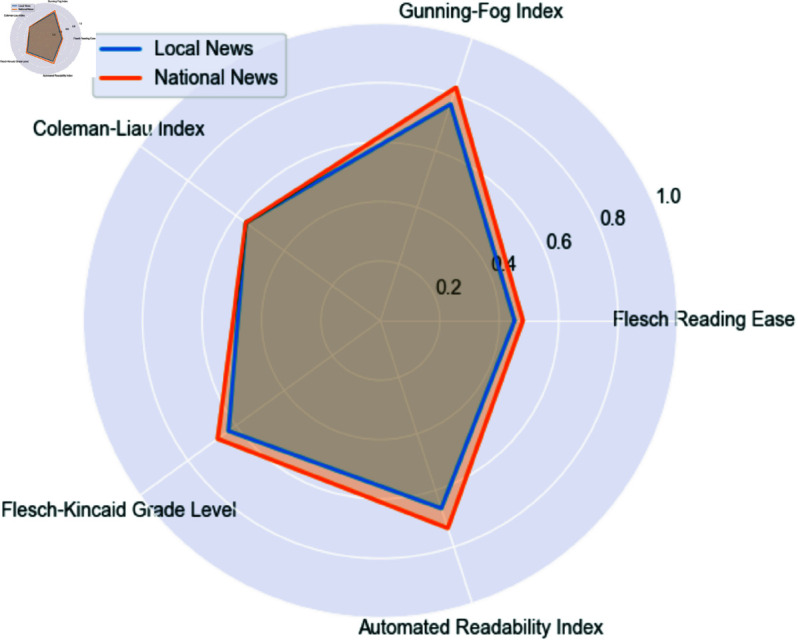
Spider chart of readability metrics in local vs. national news articles.

The readability of articles was assessed using multiple metrics, Fig 5 and Table 4. While several lexical richness metrics (such as Type-Token Ratio, Herdan’s C, Dugast’s U, MTLD, and HDD) are higher in local news articles, others (notably Yule’s K and MATTR) are higher in national news, with the differences being statistically significant. This suggests that the relationship between scale of coverage and lexical diversity is not straightforward, and no clear pattern of superiority emerges. However, when considered alongside the readability results, it is notable that both local and national news outlets appear to balance accessibility with a degree of linguistic richness. Local news, despite its generally simpler readability, still incorporates a diverse vocabulary, while national news, even as it addresses a broader audience, does not sacrifice lexical variety. This indicates that both types of outlets pursue a nuanced editorial strategy that values both clarity and expressiveness, tailored to their respective audiences. Further qualitative analysis will help clarify how these patterns manifest in practice.

**Table 4:** Descriptive statistics and Mann-Whitney U test results for readability metrics.

	Flesch Reading Ease	Gunning-Fog Index	Coleman-Liau Index	Flesch-Kincaid Grade Level	Automated Readability Index
**Descriptive Statistics (Local)**					
**Mean**	**46.14**	**13.76**	**10.07**	**11.37**	**11.95**
**Std**	**9.56**	**2.76**	**1.65**	**2.56**	**3.28**
**Min**	**0.**	**3.33**	**2.52**	**2.68**	**0.82**
**Max**	**207.**	**201.36**	**26.47**	**206.03**	**263.97**
**Descriptive Statistics (National)**					
**Mean**	**47.96**	**14.83**	**10.07**	**12.17**	**13.21**
**Std**	**10.13**	**2.93**	**1.92**	**2.67**	**3.41**
**Min**	**0.**	**3.73**	**-1.33**	**1.53**	**-2.43**
**Max**	**207.**	**57.85**	**19.60**	**52.33**	**65.13**
**Mann-Whitney U Test Results**					
**Metric**	**Test**	**Statistic**	**p-value**
Flesch Reading Ease	Mann-Whitney U	4160596591.0	*p*<0.001**		
Gunning-Fog Index	Mann-Whitney U	6168626733.0	*p*<0.001**		
Coleman-Liau Index	Mann-Whitney U	4994236123.5	0.0014**		
Flesch-Kincaid Grade Level	Mann-Whitney U	5992028447.0	*p*<0.001**		
Automated Readability Index	Mann-Whitney U	6216163585.5	*p*<0.001**		

#### 4.1.6 Lexical richness metrics.

**Fig 6:**
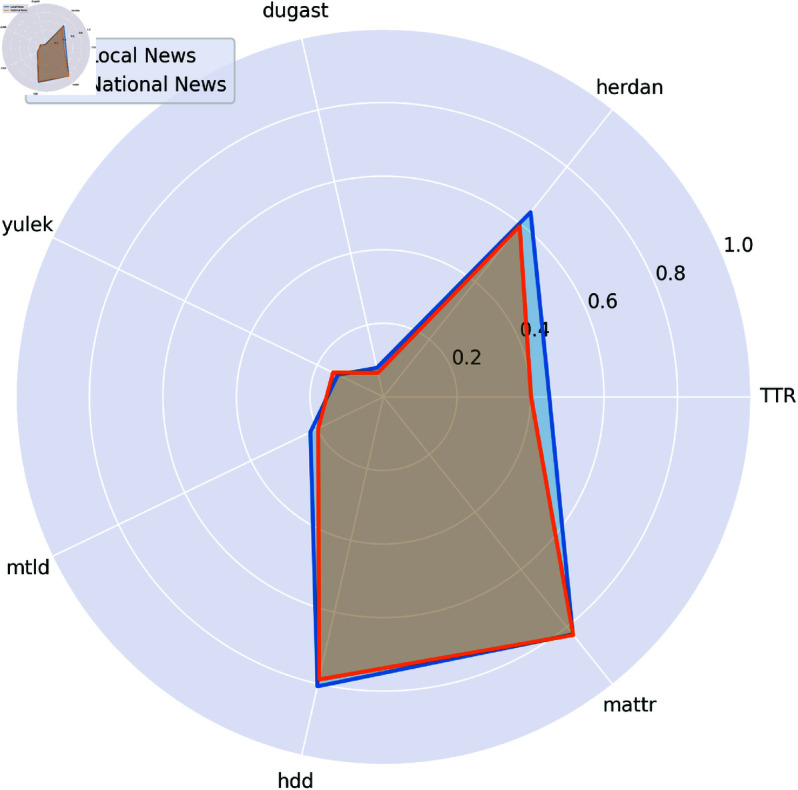
Spider chart of lexical richness metrics in local vs. national news articles.

Lexical richness was assessed using multiple indicators. In Fig 6 and Table 5, local news articles score higher in certain metrics such as Type-Token Ratio, Herdan’s C, Dugast’s U, MTLD, and HDD. By contrast, Yule’s K and MATTR are higher in national articles, although MATTR is not significant at most thresholds. These results highlight that local and national news differ in specific aspects of lexical richness that are measured by each index.

**Table 5:** Descriptive statistics and Mann-Whitney U test results for lexical richness metrics.

	TTR	Herdan	Dugast	Yulek	MTLD	HDD	MATTR
**Descriptive Statistics (Local)**							
**Mean**	**0.5524**	**0.8988**	**60.3458**	**100.0620**	**111.1106**	**0.9197**	**0.9280**
**Std**	**0.0874**	**0.0181**	**15.6068**	**32.9020**	**33.7417**	**0.0227**	**0.0217**
**Min**	**0.1843**	**0.7451**	**16.5634**	**0.0**	**9.3535**	**0.5833**	**0.5857**
**Max**	**1.0**	**1.0**	**558.4916**	**725.6236**	**470.6800**	**1.0**	**1.0**
**Descriptive Statistics (National)**							
**Mean**	**0.5118**	**0.8853**	**52.9780**	**110.3483**	**100.2689**	**0.9118**	**0.9287**
**Std**	**0.0612**	**0.0190**	**9.9502**	**31.7456**	**28.3800**	**0.0219**	**0.0187**
**Min**	**0.2308**	**0.7175**	**16.4385**	**33.2410**	**12.2667**	**0.6858**	**0.6948**
**Max**	**0.8889**	**0.9730**	**168.5176**	**473.3092**	**324.0**	**0.9701**	**0.9965**
**Mann-Whitney U Test Results**							
**Metric**	**Test**	**Statistic**	**p-value**				
TTR	Mann-Whitney U	3702371585.5	*p*<0.001**				
Herdan	Mann-Whitney U	3157190215.0	*p*<0.001**				
Dugast	Mann-Whitney U	3216407904.5	*p*<0.001**				
Yulek	Mann-Whitney U	6496266541.5	*p*<0.001**				
MTLD	Mann-Whitney U	4154895222.0	*p*<0.001**				
HDD	Mann-Whitney U	3961182192.5	*p*<0.001**				
MATTR	Mann-Whitney U	5246889948.5	0.0484*				

The higher lexical richness in local news, as measured by several indices, is an intriguing finding. It suggests that, despite simpler readability, local news employs a more varied vocabulary, likely due to the need to reference specific local terms, names, and cultural references. This challenges the assumption that local news is necessarily less sophisticated linguistically and points to a nuanced editorial balancing act: being both accessible and richly descriptive. National news, by contrast, may rely on more standardized language to ensure broad comprehensibility.

### 4.2 Topic modeling results

In this section, we present the results of the topic modeling analysis conducted using the BERTopic framework. The analysis was performed separately on the local and national news datasets, followed by a comparative evaluation.

#### 4.2.1 Hierarchical structure of topics.

**Fig 7:**
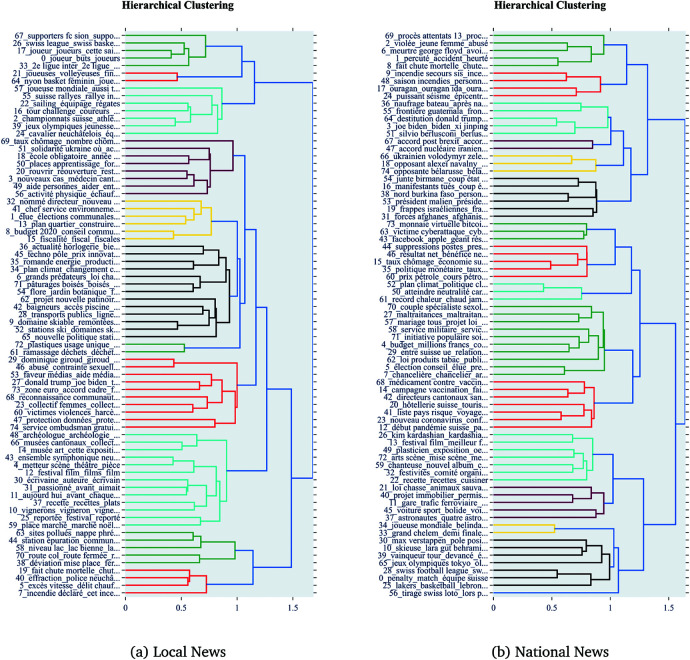
Comparison of topic modeling on local vs national news articles.

The topic modeling identified hierarchical structures of topics for both datasets. Fig 7 provides a side-by-side comparison of these hierarchical visualizations.

The hierarchical visualization reveals that local news topics are indeed organized around very local-level issues or entities, while national news topics are organized around broader themes such as national politics, international news, and major sports events. This indicates that local news tends to focus on more granular and specific local issues, whereas national news covers a wider range of themes with broader geographical and thematic scope.

#### 4.2.2 Linguistic prevalence in topics.

**Fig 8:**
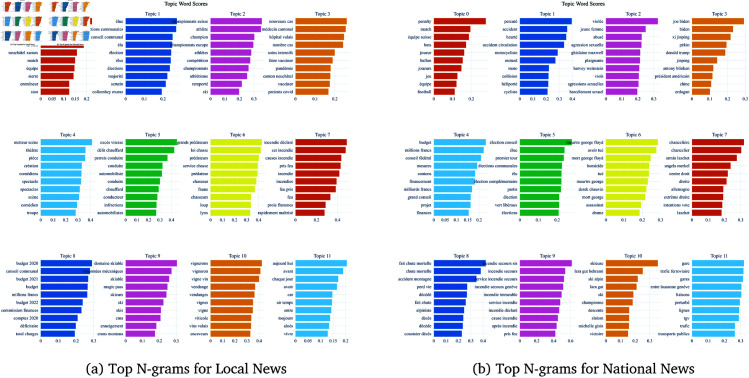
Most relevant n-grams (1-to-3-gram) for the top 12 topics for local and national news.

To further analyze the differences in topic prevalence, we examined the distribution of word prevalence within each dataset and each topic. Fig 8 illustrates the comparative distribution of topics between the local and national news datasets.

The topic distribution comparison highlights that local news shows a higher concentration on local topics, such as local football competitions or local elections, while national news covers a more diverse set of topics, including international politics and large football competitions. This suggests that local news outlets prioritize issues relevant to their immediate audience, whereas national news outlets address a broader array of topics to cater to a wider audience. These findings reinforce the theoretical distinction between local and national news agendas, as discussed in the literature [45,49]. The pronounced focus on community-specific topics in local news not only reflects editorial priorities but also shapes the contours of public discourse and civic engagement at the local level. Conversely, the broader thematic spread in national news may dilute the sense of proximity but enables coverage of issues with wider societal impact. This divergence in agenda-setting will be further examined in the qualitative analysis, where we explore how these topic choices are framed and narrated.

#### 4.2.3 Longitudinal trends.

**Fig 9:**
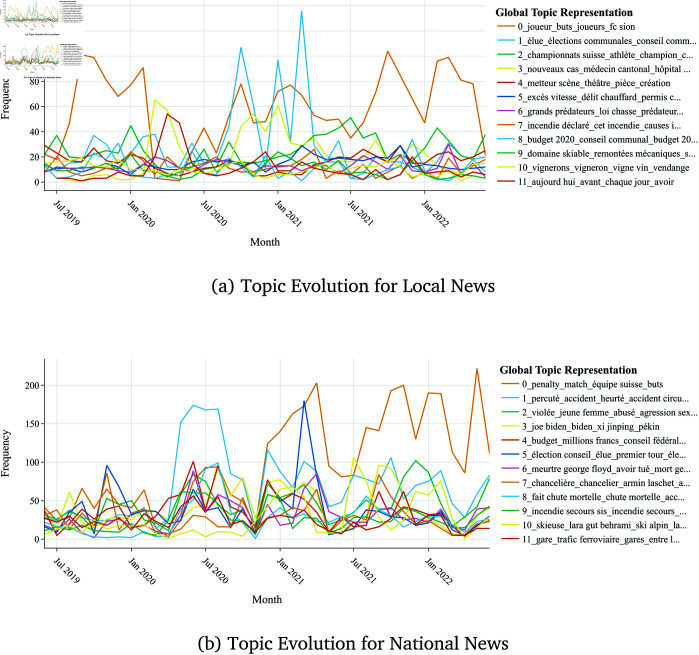
Distribution over time (monthly) of the top 12 topics for local and national News.

Over the three-year period from June 2019 to June 2022, we observed specific trends and shifts in topic prevalence. Fig 9 shows the longitudinal trends in topic prevalence for both local and national news datasets.

These longitudinal trends suggest that for both scales, the prevalence of a topic is directly linked to its topicality. Topics become more prominent in the news coverage when they align with current events or public interest. For instance, during election periods, political topics gained attention, while health-related topics surged during the pandemic. While these temporal patterns are expected, the data also reveal subtle differences in how local and national outlets respond to and sustain coverage of major events. Local news may maintain a longer focus on issues with enduring community relevance, whereas national news coverage appears more event-driven and transient. This distinction has important implications for how collective memory and local identity are constructed through media, a theme that will be unpacked in the subsequent qualitative and discussion sections.

Overall, the topic modeling results provide a comprehensive understanding of the thematic differences and similarities between local and national news, as well as the evolution of these topics over time.

### 4.3 Comprehensive analysis of selected topics

#### 4.3.1 Selection of topics.

The qualitative analysis aimed to deepen understanding of content and framing differences between local and national news. Using the topics generated by the BERTopic model 3, we selected articles within themes shared by both media scales. We defined six themes that would include multiple prevalent topics: (1) Sports, (2) Environment, Animals & Climate, (3) Political Scenes, (4) Business & Economy, (5) Cultural Events, and (6) Justice & *Faits Divers*. *Faits Divers* refers to miscellaneous news items involving local incidents, accidents, or minor crimes. These themes were selected due to their strong representation in both datasets and their presumed differential treatment between local and national media.

We conducted a stratified sampling of 100 articles for each theme, divided equally between local and national sources, and analyzed them using iterative grounded theory to identify general patterns (Table 6).

**Table 6:** Number of articles by scale, theme, and journal (with totals).

Theme	Local				Romandy			
	Arc Info	La Côte	Le Nouvelliste	**Total**	20 minutes	24 heures	La Tribune de Genève	**Total**
Sports	2,059	1,048	1,368	**4,475**	4,006	2,282	2,265	**8,553**
Environment	243	193	397	**833**	370	412	405	**1,187**
Politics	740	767	824	**2,331**	1,312	2,565	2,000	**5,877**
Economy	264	154	449	**867**	475	743	618	**1,836**
Culture	868	482	660	**2,010**	614	731	697	**2,042**
Justice	625	382	539	**1,546**	2,796	1,829	1,745	**6,370**

#### 4.3.2 Qualitative analysis.

The qualitative analysis revealed four main theories of differences between local and national news, resulting from the saturation process of the Grounded Theory. These axes are further articulated by drawing on Fairclough’s critical discourse analysis and Goffman’s frame analysis to interpret the observed patterns.

**The proportionality of temporal and geographical scales.** A key distinction between local and national news coverage lies in the proportionality between the scale of the outlet and the temporal and geographical framing of events. This dimension reveals how time and space are constructed discursively at different scales of reporting. Local newspapers consistently anchor their reporting in the immediate present and the specificities of place, foregrounding events that are temporally imminent and geographically proximate. This is evident in the way local sports coverage, for example, centers on the outcome of a single match or previews an event happening within days, often referencing particular neighborhoods, streets, or community institutions. Such discursive choices, as theorized by Fairclough, reflect a social practice in which the local press positions itself as an integral participant in the everyday life of its audience, reinforcing a sense of shared belonging and immediacy. A representative quote here might be a local article announcing, “Le FC Sion s’impose contre le Servette FC à Genève, 2-1. Les réactions après cette victoire”, which encapsulates both the temporal immediacy and the spatial specificity characteristic of local news.

In contrast, national newspapers tend to frame events within broader temporal arcs and spatial scales. Sports results are contextualized within the narrative of an entire season or national competition, and cultural events are discussed in relation to their significance for a wider public. This approach, illuminated by Goffman’s frame analysis, invites readers to adopt a more observational stance, positioning them as members of a larger imagined community rather than direct participants in a local scene. For instance, a national article might state, “Football: Servette poursuit sa route en Europe. - Plus avancé, moins convaincant, mais toujours bien grenat”, thus situating the event within an ongoing narrative that extends beyond the immediate locality.

This proportionality is not merely a matter of stylistic preference, it encodes distinct editorial ideologies about the function of journalism. Local news, by highlighting the here-and-now and the familiar, fosters direct engagement and a sense of community embeddedness, through a textual proximity. National news, by contrast, constructs a broader societal perspective, emphasizing continuity, comparison, and synthesis across time and space. The systematic use of temporal and geographical markers in each type of outlet thus reveals how scale is enacted through both language and narrative structure, shaping the reader’s relationship to the news and to the community it represents.

**Promotion of direct consumption versus advertising campaign.** A further axis of differentiation between local and national news emerges in the treatment of commercial content and the boundary between journalism and advertising. Local newspapers frequently feature articles that promote regional products, services, or events, often blurring the line between editorial content and advertisement. These pieces are typically anchored in the concrete realities of the community, naming specific artisans, local businesses, or venues, and providing detailed descriptions that serve both an informative and promotional function. From a Faircloughian perspective, this discursive practice reflects the embedding of economic and social practices within the text: the local press not only reports on community life but actively participates in the economic circulation of the locality, reinforcing its role as a facilitator of local commerce and social cohesion. A representative quote might be a local article stating, “Plus de vingt vignerons accueilleront samedi les amateurs de crus sur leur domaine. Au coeur des vendanges\guillemotright permettra de découvrir le métier et le quotidien des vignerons”, which exemplifies the intertwining of information, place, and direct consumption.

National newspapers, by contrast, tend to integrate commercial content within broader advertising campaigns or brand narratives. Rather than promoting a specific product or event for immediate consumption, national outlets often feature interviews with cultural figures, coverage of corporate strategies, or previews of major releases, situating these within long-term storytelling that aligns with the interests of a wider, more heterogeneous audience. Goffman’s frame analysis helps elucidate this distinction: national news frames consumption as part of a larger narrative, be it the trajectory of a brand, the anticipation of a film’s release, or the evolution of a market, inviting readers to engage as spectators in ongoing economic and cultural performances. For example, a national article might read, “Alors qu’il prépare une nouvelle version de «Premiers baisers», Jean-Luc Azoulay songe à reprendre une autre de ses séries des années 1990”, thus positioning the event within a broader commercial spectacle and a longer-term connection with its audience rather than a direct call to local action.

This divergence in commercial framing reflects deeper editorial ideologies about the relationship between journalism, community, and the marketplace. Local news, by emphasizing direct consumption and community participation, reinforces its integration in the local social fabric and its responsiveness to the immediate needs and interests of its audience. National news, by contrast, constructs consumption as a mediated, collective experience, emphasizing trends, reputations, and narratives that transcend the local. The systematic use of local anchorage and direct appeals in local news, versus the narrative integration and brand focus in national news, thus reveals how each outlet negotiates its dual role as both informer and economic actor within its respective sphere.

**Pedagogy and popularization of technicality in contrast with direct communication.** The ways in which local and national newspapers handle complex or technical subjects reveal distinct editorial philosophies and communicative strategies. National outlets, when reporting on topics such as economics, science, or the justice system, frequently draw on the authority of experts and institutional data. Their articles often weave together specialized terminology, statistical evidence, and interpretive commentary, constructing a narrative that both signals credibility and demands a certain level of reader detachment. They address a more heterogeneous and less regular audience. This approach, illuminated by Fairclough’s critical discourse analysis, situates the national press as a conduit for institutional knowledge, mediating between the expert sphere and the lay public. Yet, these same articles often include efforts to bridge the gap, such as sidebars or links that break down terminology, or explanatory passages that translate numbers into implications for everyday life. For instance, a national news piece might read, “Le PMI industriel s’est apprécié de cinq points sur un mois et de près de 23 points sur un an pour s’établir à 66,3 points, loin au-delà des 50 points marquant le seuil de croissance. (...) L’embellie dans l’industrie est plus marquée encore que ne l’escomptaient les économistes sondés par AWP”. Here, the technical data is both presented and interpreted, inviting the reader to understand its broader significance.

Local newspapers, on the contrary, tend to avoid abstraction in favor of immediacy and clarity. Their coverage of new policies, scientific findings, or legal changes is typically grounded in the practical realities of the community. In addition to foregrounding expert authority, local journalists focus on how such developments will affect readers’ daily lives, often using plain language and concrete examples. This communicative stance, as Goffman’s frame analysis suggests, positions the audience not as passive recipients of distant expertise, but as active members of a shared social world. A typical local article might state, “L’animal aperçu d’abord dans la région de Grimisuat puis à Bramois est bien un loup. Le Service de la chasse attend les résultats d’analyses pour l’identifier et déterminer si l’individu est déjà répertorié dans notre canton”. The emphasis is on actionable information, delivered in a tone that assumes familiarity and shared interest.

These contrasting editorial choices actively delineate the boundaries of journalistic responsibility and audience engagement, more than shaping the tone of reporting. National news, by oscillating between technicality and popularization, asserts its role as an interpreter of complex systems for a broad, sometimes anonymous audience. Local news, in privileging directness and relevance, underscores its commitment to serving as a practical resource and a voice within the community, a “service journalism” provider. The editorial choices around language, authority, and explanation thus become key sites where the social function of each media scale is negotiated and enacted.

**Narrative coherence balanced with informational objectives.** The interplay between storytelling and information delivery marks another salient difference between local and national news. Local journalism often gravitates toward narratives that foreground collective experience and community solidarity. Stories are constructed to resonate with shared values and local identity, sometimes at the expense of exhaustive factual detail. This tendency is visible in coverage of local celebrations, sports victories, or incidents, where the narrative arc emphasizes togetherness, mutual support, or the perseverance of the community. Fairclough’s perspective on discourse as a vehicle for social meaning is instructive here: the local press does not just recount events, but actively shapes a sense of belonging through the selection and arrangement of narrative elements. For example, a local report on a sports triumph might highlight the contributions of coaches, volunteers, and supporters, weaving individual actions into a tapestry of communal achievement. A quote such as, “Joël Magnin n’a de cesse de le répéter, la force de son équipe réside avant tout dans son abnégation et sa volonté de se surpasser pour le coéquipier”, would encapsulate this collective framing.

National outlets, meanwhile, tend to privilege narrative structures that spotlight individual agency, dramatic developments, or broader societal implications. Their storytelling often orbits around prominent figures, exceptional cases, or emblematic events, using these as entry points to explore larger trends or issues. Goffman’s frame analysis helps clarify this orientation: national news frames the audience as observers of noteworthy stories, inviting reflection or debate rather than direct identification. In the context of crime or legal proceedings, for instance, national coverage may focus on the spectacle of a celebrity trial or the intricacies of a high-profile scam, constructing a narrative that is both engaging and detached from everyday experience. A typical national article might assert, “Un escroc espagnol a été condamné ce lundi pour avoir recueilli plus de 260’000 euros afin de financer un prétendu traitement expérimental”, thus situating the event within a broader, sometimes critical, societal lens.

These divergent narrative logics are rooted in deeper editorial priorities and assumptions about the reader’s relationship to the news. Local journalism, by privileging coherence and shared meaning, fosters a participatory ethos and reinforces the press’s role as a communal institution. National journalism, in contrast, leverages narrative to contextualize and interpret, positioning itself as a guide through the complexities of public life. The balance each outlet strikes between narrative and information thus reveals much about its vision of journalism’s purpose and its imagined audience.

#### 4.3.3 Integration with quantitative results.

The integration of quantitative metrics and qualitative analysis in this study provides a multi-layered understanding of how local and national newspapers in Switzerland construct proximity and community engagement. By systematically linking computational indicators to discourse-theoretical frameworks, we move beyond surface-level description to reveal the mechanisms by which media scale shapes narrative strategies and audience relationships.

Quantitative findings on readability and lexical richness, see Table 9, directly support Fairclough’s notion of discursive practice, where language choices reflect and reinforce social roles. Local news articles exhibit lower readability scores, indicating simpler sentence structures and greater accessibility. This aligns with our qualitative observation that local outlets prioritize narrative coherence and direct communication, fostering inclusivity and shared understanding within the community. In contrast, national news demonstrates higher readability indices, consistent with more complex syntactic constructions and specialized vocabulary. This complexity, as theorized by Fairclough, signals the national press’s role as a mediator of institutional knowledge, addressing a broader and more heterogeneous audience.

Lexical richness metrics further highlight these dynamics. Local news displays higher values in indices such as Type-Token Ratio and Herdan’s C, reflecting the use of diverse, context-specific vocabulary. This supports the qualitative finding that local outlets embed their reporting in the lived realities of their audience, referencing local figures, places, and events, see Table 8. The presence of unique local terms and names not only increases lexical variety but also enacts Goffman’s concept of frame alignment, anchoring the narrative in the immediate social world of the reader. National news, by contrast, employs more standardized language, facilitating broader comprehension but at the cost of local specificity.

The quantitative analysis of quotations, in Table 7, reveals that local articles contain more and longer direct quotes, a pattern that complements the qualitative insight that local journalism foregrounds community voices. This editorial choice exemplifies Fairclough’s social practice dimension, as the local press actively constructs a participatory public sphere by amplifying the perspectives of residents, officials, and local actors. National outlets, while still incorporating direct speech, tend to privilege expert commentary and institutional sources, reflecting a more observational and less participatory framing in line with Goffman’s analysis of audience positioning.

**Table 7:** Statistical comparison of quotes in local and national articles.

Category	Local		National		p-values	
	Count	Length	Count	Length	Quote Count	Quote Length
Sports	4.92	92.97	2.40	60.68	*p*<0.001**	*p*<0.001**
Environment	4.85	88.52	3.93	74.14	*p*<0.001**	*p*<0.001**
Politics	4.80	77.18	5.28	66.79	*p*<0.001**	*p*<0.001**
Economy	5.25	91.21	3.14	63.94	*p*<0.001**	*p*<0.001**
Culture	7.38	71.94	7.09	49.21	0.1424	*p*<0.001**
Justice	2.24	51.23	3.14	42.08	*p*<0.001**	*p*<0.001**

**Table 8:** Top 5 named entities for Sports & Cultural Events across news types and entity categories.

Rank	Event		Location		Organization	
	**Local News**	**National News**	**Local News**	**National News**	**Local News**	**National News**
**Sports**						
1	Coupe de Suisse (714)	Jeux Olympiques (1356)	Sierre (1254)	Tokyo (1676)	HCC (1909)	Servette (1609)
2	Play-off (674)	Finale (996)	La Chaux-de-Fonds (1029)	Suisse (1512)	FC Sion (1660)	NBA (1567)
3	Championnat (518)	Play-off (864)	Suisse (831)	Lausanne (1507)	NUC (1241)	NHL (872)
4	Match (464)	Ligue des champions (613)	Nyon (825)	Genève (1505)	Union neuchâtel (881)	Grand chelem (714)
5	Derby (277)	Coupe du monde (595)	Sion (784)	Pékin (816)	Xamax (867)	CIO (614)
**Cultural Events**						
1	Festival (240)	Oscars (205)	Neuchâtel (1000)	Genève (436)	Conseil fédéral (122)	Netflix (448)
2	Pandémie (161)	Pandémie (157)	La Chaux-de-Fonds (771)	Lausanne (415)	NIFFF (111)	ATS (91)
3	Exposition (88)	Festival de Cannes (104)	Sion (416)	Suisse (329)	Visions du réel (91)	HBO (85)
4	Vernissage (73)	Festival (100)	Valais (409)	France (290)	Confédération (83)	Conseil Fédéral (82)
5	Manifestation (70)	Golden Globes (98)	Nyon (390)	Paris (269)	RTS (75)	Académie (76)

**Table 9:** Comparison of readability and lexical richness metrics between local and national news for environment and justice Topics.

Environment, Animals & Climate			
*Readability Metrics*			
**Metric**	**Local Mean**	**National Mean**	**p-value**
Flesch Reading Ease	45.61	46.43	0.0509
Gunning-Fog Index	14.29	14.75	*p*<0.001**
Coleman-Liau Index	10.10	10.30	0.0150*
Flesch-Kincaid Grade Level	11.48	11.68	0.0709
Automated Readability Index	12.07	12.85	*p*<0.001**
*Lexical Richness Metrics*			
**Metric**	**Local Mean**	**National Mean**	**p-value**
Type-Token Ratio	0.55	0.51	*p*<0.001**
Herdan’s C	0.90	0.88	*p*<0.001**
Dugast’s U	59.23	51.56	*p*<0.001**
Yule’s K	105.71	118.39	*p*<0.001**
MTLD	106.63	95.15	*p*<0.001**
Hypergeometric HDD	0.92	0.91	*p*<0.001**
Moving-Average TTR	0.93	0.92	0.0127*
Justice & Faits divers			
*Readability Metrics*			
**Metric**	**Local Mean**	**National Mean**	**p-value**
Flesch Reading Ease	43.15	43.84	0.0081*
Gunning-Fog Index	13.70	13.98	*p*<0.001**
Coleman-Liau Index	9.43	9.45	0.6977
Flesch-Kincaid Grade Level	10.90	11.13	0.0012**
Automated Readability Index	11.09	11.80	*p*<0.001**
*Lexical Richness Metrics*			
**Metric**	**Local Mean**	**National Mean**	**p-value**
Type-Token Ratio	0.57	0.50	*p*<0.001**
Herdan’s C	0.90	0.87	*p*<0.001**
Dugast’s U	53.10	46.68	*p*<0.001**
Yule’s K	122.10	126.18	*p*<0.001**
MTLD	87.44	87.11	0.6717
Hypergeometric HDD	0.90	0.90	*p*<0.001**
Moving-Average TTR	0.92	0.93	*p*<0.001**

Named entity recognition, in Table 8, further substantiates these distinctions. Local news is characterized by frequent references to hyper-local entities, such as specific neighborhoods, local organizations, and community leaders, whereas national news features a higher prevalence of national and international actors. This pattern operationalizes the theoretical distinction between proximity as a discursive resource from Fairclough and as a framing device from Goffman, showing how editorial scale is enacted through the selection and emphasis of entities.

Finally, topic modeling and longitudinal analysis, see Figs 7 and 9, reveal that local news maintains sustained attention on community-relevant issues, while national news coverage is more event-driven and thematically diffuse. This supports the qualitative finding that local outlets construct collective memory and identity through ongoing engagement with recurring local themes, whereas national outlets frame news as part of broader societal narratives.

In sum, the integration of quantitative and qualitative results demonstrates that the observed differences between local and national news are more than stylistic preferences, but reflect deeper editorial ideologies and social functions. By grounding computational metrics in discourse and frame theory, this study provides a novel, analytically rich account of how media scale shapes the construction of proximity, identity, and public engagement in the Swiss context. This comprehensive approach sets the stage for a theoretically informed discussion of the implications for media practice and democratic participation.

## 5 Discussion

### 5.1 Interpretation of the findings

This study advances the understanding of how local and national newspapers in Romandy, Switzerland, construct proximity and community through distinct narrative strategies, offering several nuanced contributions to the literature on local journalism, media discourse, and democratic engagement.

**Nuanced contributions to the literature.** First, while previous research has sometimes found minimal differences in framing between local and national outlets on high-salience issues [45,61], our analysis reveals that, even when covering similar events, local and national newspapers in Romandy diverge in their narrative construction and temporal framing. Local outlets foreground immediacy, community relevance, and shared local knowledge, whereas national outlets contextualize events within broader, often more abstract, national or international narratives. This adds nuance to the assumption of homogeneity in news framing across scales, as discussed by Lawlor [45] and Funk & McCombs [49].

Second, whereas much of the literature has conceptualized proximity primarily in terms of geographic coverage or audience reach [8,10,11], our findings demonstrate that proximity is also actively constructed through linguistic and narrative devices. Local outlets in Romandy employ direct quotations, references to local places and people, and implicit cultural cues, thereby fostering a sense of community belonging and shared identity. This supports and extends the work of Pignard-Cheynel and Amigo [43], who highlight participatory practices and audience inclusion in local news, and aligns with Goffman’s [81] notion of frame alignment as a means of making news meaningful to specific communities.

Third, our results challenge the narrative of inevitable decline in local journalism. While prior studies have linked the erosion of local news to increased polarization and diminished community cohesion [10,65,66], we find that local outlets in Romandy continue to prioritize community engagement and participatory practices, even under economic strain. This resilience and adaptability, also observed in recent European studies [11,12], suggest that local journalism retains a distinct and vital role in shaping local identity and democratic participation.

Fourth, by integrating readability and lexical richness metrics into our analysis, we move beyond the focus on topic prevalence or sentiment that characterizes much computational research [18,19]. Our findings reveal that local news is not simply “simpler” or “less sophisticated” than national news; rather, it is tailored to its audience’s needs, with higher lexical richness in some domains and greater accessibility in others. This reflects a nuanced adaptation to community audiences, challenging the notion of local news as a “scaled-down” version of national coverage.

Finally, our mixed-methods pipeline demonstrates the value of integrating computational and qualitative approaches. By combining topic modeling, named entity recognition, and discourse analysis, we provide a more comprehensive account of how local and national news construct proximity and community identity, building on calls for methodological innovation in media studies [23,49].

**Theoretical integration.** The application of Fairclough’s critical discourse analysis framework [58] enables us to interpret these findings at multiple levels. At the textual level, local media’s use of familiar references and direct speech exemplifies how language choices construct proximity and inclusion. At the discursive practice level, the participatory and interactive features observed in local news reflect evolving production and consumption practices, as local outlets increasingly engage audiences through participatory journalism and community-driven content [19,43]. At the social practice level, these narrative strategies reinforce local identities and democratic participation, as evidenced by the correlation between local media engagement and voter turnout [44,68].

Goffman’s concept of frame alignment [81] further elucidates how local and national media construct meaning for their respective audiences. Local newspapers frame events in ways that resonate with community norms and expectations, while national outlets employ frames that situate events within broader societal or political contexts. This duality underscores the complementary roles of local and national media in mediating between individual experience and collective understanding.

**Novelty and implications.** A key contribution of this study lies in its mixed-methods approach, which enables a systematic comparison of narrative strategies across media types and over time. The longitudinal analysis reveals not only differences in thematic focus and agenda-setting, but also the temporal dynamics of issue salience, for example, the distinct ways in which local and national outlets respond to media events such as elections or public health crises. These findings move beyond prior work by demonstrating that local and national media do not merely differ in content, but also in the temporal rhythms and narrative forms through which they engage their audiences.

Moreover, our results highlight the adaptive strategies of local media in response to digital transformation and changing audience expectations. The emphasis on participatory practices and community engagement observed in Romandy mirrors broader trends in local journalism across Europe and North America [7,11]. This suggests that local newspapers, despite resource constraints, continue to play a vital role in sustaining democratic engagement and fostering social capital at the community level.

**Broader significance and future directions.** In sum, our study provides a more complex and differentiated picture of local and national news production in Romandy, Switzerland. By explicitly analyzing narrative strategies, linguistic devices, and participatory practices, we show that local journalism is not simply surviving but actively adapting to new challenges and audience expectations. These findings have important implications for understanding the resilience of local media, the construction of community identity, and the maintenance of democratic engagement in the digital age.

Future research could further explore the interplay between local and national agendas, the impact of digital platforms on narrative strategies, and the implications for democratic participation among different demographic groups, including youth [68]. Our study thus lays the groundwork for ongoing inquiry into the evolving roles of media in contemporary society.

### 5.2 Limitations

While this study offers new insights into the narrative strategies and community engagement practices of local and national newspapers in Romandy, several limitations should be acknowledged. First, the analysis is context-specific, focusing on French-speaking Switzerland, as such, the findings may not be directly generalizable to other linguistic regions or media systems with different institutional, cultural, or economic contexts [10,11]. Furthermore, the data may not be representative of other regions, given the particular context of Romandy and the affiliation of certain news sources, such as *TDG* and *24h*, with the TX Group. This ownership structure may influence editorial policies, potentially limiting the generalizability of the findings. Second, although our mixed-methods approach integrates computational and qualitative analyses, the selection of indicators and interpretive frameworks inevitably involves subjective choices that may influence the results. In particular, the content analysis was conducted by a single annotator, which may affect the reliability of the qualitative findings. To mitigate this issue, a methodical approach was employed, enabling readers to assess the relevance and robustness of the findings. Third, while we have demonstrated the discursive construction of proximity, our study is limited to textual analysis and does not include audience reception data or ethnographic observation, which could further illuminate how these narratives are received and interpreted by different community groups [43,68]. Fourth, data access for local news was subject to certain restrictions, potentially limiting the completeness or representativeness of the sample. Finally, the temporal scope of the study, while sufficient to capture major events and trends, may not account for longer-term shifts in media practices or audience engagement. Ultimately, the observed differences may be influenced by the specific editorial policies of the media outlets studied. However, the analysis aligns with existing studies on local and national media comparisons, suggesting that these findings reflect broader trends, while also providing new nuances that enhance the interpretation of press dynamics. Future research could address these limitations by expanding the comparative scope to include other regions or countries, incorporating audience studies, and exploring the impact of digital platforms and social media on the construction of proximity and community identity.

## 6 Conclusion

This study set out to systematically compare the discursive construction of community proximity in local and national newspapers in Romandy, Switzerland, through a novel mixed-methods pipeline that integrates computational linguistic metrics with qualitative discourse and frame analysis. Our approach addressed two central research questions:

- RQ1: How can a mixed-methods analytical pipeline, combining computational linguistic metrics and qualitative discourse analysis, be systematically designed and applied to uncover the discursive construction of community proximity in digital news content?
- RQ2: In what ways do local and national newspapers in Romandy, Switzerland, differ in their narrative strategies, thematic focus, and engagement with community identity, as revealed by this integrated approach? How do these differences reflect the distinct roles and societal functions of local versus national media, as interpreted through critical discourse and frame analysis?

To address RQ1, we developed and validated a comprehensive, reproducible pipeline that bridges large-scale computational analysis with grounded qualitative interpretation. This methodological innovation enables the systematic identification of narrative strategies and discursive practices across vast news corpora, setting a precedent for future interdisciplinary research in journalism studies.

In response to RQ2, our comparative analysis revealed that local and national newspapers in Romandy deploy fundamentally distinct editorial logics. Local outlets foreground immediacy, personalization, and community anchorage, employing narrative coherence, direct quotations, and references to familiar people and places to foster a sense of belonging and shared identity. In contrast, national outlets adopt broader temporal and spatial frames, privileging contextualization, technical vocabulary, and expert commentary to address a heterogeneous audience and situate events within wider societal narratives.

By explicitly integrating Fairclough’s critical discourse analysis and Goffman’s frame analysis, our findings move beyond descriptive comparison. We demonstrate that proximity is not only a function of geographic scale, but is also actively constructed through linguistic, narrative, and framing devices. Local news enacts proximity through participatory and inclusive discourse, while national news constructs a more observational and interpretive stance, mediating between individual experience and collective understanding.

The novelty of this study lies in its analytical depth and theoretical integration. Our results show that even when covering similar events, local and national outlets diverge in their narrative construction, temporal rhythms, and engagement strategies. This challenges assumptions of homogeneity in news framing and highlights the resilience and adaptability of local journalism in the face of economic and technological pressures. The integration of computational and qualitative methods provides a richer, more nuanced account of how media scale shapes public discourse, community identity, and democratic participation.

These insights have significant implications for both media theory and practice. They underscore the importance of supporting local journalism as a cornerstone of democratic life, while recognizing the complementary role of national media in fostering societal integration. Our findings also suggest that the discursive construction of proximity and identity remains a vital site of innovation and contestation in the evolving media landscape.

Despite the study’s contextual and methodological limitations, it opens new avenues for research. Future work could extend this approach to other regions, languages, or media systems, incorporate audience reception studies, and further explore the impact of digital platforms on narrative strategies and democratic engagement.

In sum, this research advances the ongoing dialogue on the complex dynamics between local and national news, offering a robust analytical framework and new empirical evidence on the construction of proximity, identity, and public engagement. As the media environment continues to transform, such comprehensive, theory-driven analyses are essential for understanding and sustaining the social fabric that connects communities to the broader public sphere.

## Supporting information

S1 Appendix Illustration of content analysis methodology. See S1 Appendix for a detailed description of the qualitative coding process.
(PDF)
