# Supplementary material for: Decoding community proximity discourse: A mixed-methods comparative analysis of online local and national newspapers in Romandy, Switzerland
Source: PLoS One. 2025 Aug 1;20(8):e0328059. doi: 10.1371/journal.pone.0328059 (PMC12316251; doi:10.1371/journal.pone.0328059)
Supplement: S1 Appendix — See S1 Appendix for a detailed description of the qualitative coding process. (PDF) [file pone.0328059.s001.pdf]

S1 Appendix. Illustration of content analysis methodology

1 S1: Appendix - Illustration of content analysis methodology

This appendix illustrates the content analysis methodology applied in our study, focusing on the qualitative analysis of local and national news in Romandy, Switzerland. The analysis was conducted using a grounded theory approach, incorporating the Fairclough model and Goffman framework, as detailed in the main text. Here, we outline the process of annotation and coding that led to the development of the four main theoretical axes described in the qualitative analysis section.

1.1 Theoretical frameworks

The Fairclough model and Goffman frame analysis were applied due to their utility in analyzing media discourse and social interactions. The Fairclough model of critical discourse analysis facilitates the examination of how language constructs social realities. Goffman's frame analysis provides insights into how news stories are organized through interpretive frames, highlighting which aspects of events are emphasized, how issues are contextualized, and what perspectives are foregrounded.

1.2 Open coding

The initial phase of open coding involved a thorough examination of the news articles to identify and categorize distinct elements. During this phase, various linguistic and narrative features were annotated, including:

- **Language**: Identification of meliorative and pejorative propositions, jargon, quotations, and tone indicators. For instance, local news often employed meliorative language to promote local cultural events and producers, while national news utilized jargon and a serious tone, particularly in economic and scientific contexts. The Fairclough model facilitated this analysis by highlighting how language choices contribute to the construction of social realities and the promotion of certain ideologies.
- **Narratives**: Types of arguments, contextualization elements, and depoliticization were annotated. Local news narratives frequently focused on community-centric stories, whereas national news narratives included broader, depoliticized contexts. Goffman's frame analysis allowed examination of how narratives are framed to present certain facets of society, such as nature being portrayed as a spectacle removed from the immediate human environment.
- **Scope**: Geographical and temporal elements were noted, such as the strong local anchorage in local news with references to specific streets and municipalities, and the broader temporal context in national news, like entire sports seasons. This aligns with Goffman's concepts of framing and the organization of experience.
- **Stakeholders**: Identification of individuals, institutions, groups, and organizations. Local news often highlighted community figures and local institutions, while national news featured larger organizations and well-known personalities. The Fairclough model assisted in understanding how different stakeholders are represented and how power relations are communicated through media texts.

1.3 Axial coding

In the axial coding phase, relationships between the categories established during open coding were identified. This involved linking annotated elements to broader themes and patterns, while incorporating intermediary elements that emerged during the analysis:

- **Temporal and Geographical Scales**: The proportionality of temporal and geographical scales was evident, with local news focusing on immediate events and national news providing broader context. Intermediary elements such as references to specific municipalities and short temporal periods in local news highlighted the proximity relationship with readers. In contrast, national news contextualized sports performances within entire seasons or competitions.
- **Consumption and Advertising**: The distinction between direct consumption promotion in local news and integrated advertising campaigns in national news emerged. Local articles often blurred the line between news and advertisement, using strong local anchorage like naming streets and nearby stores. The Fairclough model was used to analyze these embedded marketing strategies, revealing how language is employed to subtly promote local businesses. National articles participated in long-term brand storytelling, using puns in titles for movie ad campaigns and emphasizing stable economic performance as a branding strategy for larger companies.
- **Pedagogy and Communication**: The contrast between the pedagogical approach of local news and the technical communication of national news was highlighted. Local news simplified complex topics, often involving experts to explain laws and provide practical information to residents. In contrast, national news employed a serious tone with extensive use of statistics and jargon, particularly in economic and scientific articles, aiming to address a broader audience.
- **Narrative Coherence and Informational Objectives**: The balance between narrative coherence and informational objectives was observed, with local news focusing on community solidarity and national news on individual achievements and unusual stories. Intermediary elements included the contemplative relationship to nature in local news, with stories of wildlife and preservation areas. Utilizing Goffman's frame analysis, this portrayal was interpreted as positioning nature as a spectacle, fostering a distant and observational relationship rather than an interactive one. In national news, there was a tendency to personify pets and discuss distant catastrophes. Additionally, local news often detailed accidents with a focus on responsibility, while national news highlighted tales of banditry and celebrity trials.

1.4 Selective coding

The final phase of selective coding involved refining the core categories and integrating them into cohesive theories. This process led to the development of the four main axes of differences between local and national news:

- **Proportionality of Scales**: The relationship between the scale of news coverage and the temporal and geographical context was solidified, emphasizing the local press's immediacy and the national press's broader scope.
- **Promotion and Advertising**: The theory of direct consumption versus advertising campaigns was crystallized, highlighting the local press's focus on immediate community interests and the national press's strategic brand narratives. The Fairclough model was utilized to dissect the discourse structures that embed marketing messages within news content.
- **Pedagogy and Technicality**: The pedagogical approach of local news contrasted with the technicality of national news, underscoring the different audience engagement strategies.
- **Narrative Coherence**: The prevalence of narrative coherence over informational objectives was confirmed, revealing biases in the portrayal of nature, accidents, and cultural practices. Applying Goffman's frame analysis allowed interpretation of these narratives as performances that shape readers' perceptions, particularly how nature is framed as an external entity for observation rather than interaction.

1.5 Interpretation of findings

The application of the Fairclough model and Goffman frame analysis provided lenses through which to conduct the analysis and interpret the findings. The Fairclough model's focus on critical discourse analysis enabled the identification of underlying power dynamics and ideological constructs within media texts. For example, the identification of marketing campaigns and the subtle advertisement of local businesses in local news articles illustrated how language is employed to influence consumer behavior.

Goffman's frame analysis allowed analysis of how news narratives construct interpretive frames for readers. The depiction of nature as a distant spectacle in both local and national news demonstrates how media frames environmental topics, often fostering a passive relationship between readers and the natural world. This aligns with Goffman's concept of front-stage performances, where certain aspects are highlighted to shape audience perceptions.

By integrating these theoretical frameworks, the understanding of the distinct characteristics of local and national news was deepened. The Fairclough model highlighted how language constructs social realities and influences consumer practices, while Goffman's frame analysis elucidated the presentation and framing of narratives within the media. These insights contribute to the broader discourse on media influence and the construction of societal norms through news reporting.

Through this systematic coding process and the application of the Fairclough model and Goffman frame analysis, meaningful insights into the distinct characteristics of local and national news were derived, as detailed in the qualitative analysis section of the paper. This appendix illustrates the methodology employed in the study and explains the theoretical underpinnings that guided the interpretation of the findings.
